# Hair cortisol, social support, personality traits, and clinical course: differences in schizophrenia and bipolar disorder

**DOI:** 10.1002/brb3.2412

**Published:** 2021-11-13

**Authors:** Fuzhong Yang, Xiangfei Hong, Jing Tao, Yupeng Chen, Yanbo Zhang, Hua Xiao

**Affiliations:** ^1^ Shanghai Mental Health Center Shanghai Jiaotong University School of Medicine Shanghai China; ^2^ Department of Psychiatry Faculty of Medicine and Dentistry University of Alberta Edmonton Alberta Canada; ^3^ State Key Laboratory of Microbial Metabolism Joint International Research Laboratory of Metabolic and Developmental Sciences School of Life Sciences and Biotechnology Shanghai Jiao Tong University Shanghai China

**Keywords:** bipolar disorder, childhood trauma, hair cortisol concentration, schizophrenia, social support, stressful life events

## Abstract

**Objective:**

This study aimed to investigate the differences in the relationship between hair cortisol concentration (HCC) and psychosocial stress, social support, clinical features, clinical course, and outcome in schizophrenia and bipolar disorder.

**Methods:**

A total of 109 schizophrenia patients, 93 bipolar disorder patients and 86 healthy controls between 18 and 60 years old were enrolled in the study. Linear regression and factor analysis were employed to examine and compare the relationship between HCC and childhood trauma, the number of stressful life events, the amount of social support in the three months before the hair cortisol assessment, clinical fearures, clinical course, and outcome in schizophrenia and bipolar disorder.

**Results:**

HCC is significantly associated with clinical syndromes, including depression–anxiety factor of Positive and Negative Syndrome Scale in schizophrenia patients, and thought disorder in bipolar disorder patients. However, HCC is positively related to social support and personality traits only in schizophrenia patients but not in bipolar disorder patients. Factor analysis indicates schizophrenia and bipolar disorder share a very similar but somewhat different structure in terms of HCC, psychosocial stress, social support, clinical features, clinical course, and outcome.

**Conclusion:**

Findings support that schizophrenia and bipolar disoder have a significant overlap in both clinical characteristics and enviromental risk factors. Aberrant HCC contributes to the complexity of clinical characteristics mainly in schizophrenia.

## Abstract 2

- HCC is associated with the severity of clinical syndromes in schizophrenia and bipolar disorder.
- HCC is related to social support and personality traits in schizophrenia but not in bipolar disorder.
- Schizophrenia and bipolar disorder have a significant overlap in clinical characteristics.

## 1 INTRODUCTION

Schizophrenia and bipolar disorders are two severe mental illnesses that have overlapped genetic risks and clinical manifestations, but distinctive disease course and outcome (Cardno & Owen, 2014). Clinical dimension analyses suggest that schizophrenia is prominent in positive, negative, depressive, excitement, and cognitive domains and the deficits of bipolar disorder are in depressive, excitement, psychotic, and cognitive domains (Allardyce et al., 2007; Dikeos et al., 2006). Neurocognitive dysfunction is common in schizophrenia and bipolar disorder, but more severe and propounding in schizophrenia than in bipolar disorder (Lewandowski et al., 2011). Schizotypal personality traits predict lifetime psychosis severity in schizophrenia and bipolar disorder, and high extraversion personality traits predict future onset of bipolar disorder (Lonnqvist et al., 2009; Urosevic et al., 2019). Bipolar disorder is recurrent and episodic. In contrast, schizophrenia's symptoms are often chronic with a fluctuated course (Aykut et al., 2017).

Psychosocial stressors are critical predisposing and precipitating factors in schizophrenia and bipolar disorder (Gallagher et al., 2016; Miklowitz, 2011). Childhood trauma and adulthood stressful life events (SLE) increase the risk of developing schizophrenia and bipolar disorder (Altman et al., 2006; Muenzenmaier et al., 2015) and trigger relapses in both disorders (Fallon, 2009; Lex et al., 2017), which reflects a stress‐vulnerability model. On the contrary, increased subjective social support helps individuals to cope with critical situations and protects them against the negative impacts of SLE on brain structures in schizophrenia (Karanci et al., 2017) and bipolar disorder (Altman et al., 2006). Usually, a higher relapse rate in bipolar disorder has been reported than in schizophrenia (Ayano & Duko, 2017). Yet, we do not know how psychosocial stressors and social support contribute to the relapse rate differences between schizophrenia and bipolar disorder.

Patients with schizophrenia and bipolar disorder often have abnormal hypothalamus–pituitary–adrenal (HPA) axis functions and cortisol levels, which indicates a neural stress‐diathesis model. Cortisol, the inner indicator of psychosocial stress, is associated with clinical features in schizophrenia and bipolar disorder (Girshkin et al., 2014). However, studies have reported high or normal morning cortisol levels in schizophrenia and bipolar disorder compared to healthy controls (Bradley & Dinan, 2010; Gallagher et al., 2007; Havermans et al., 2011). These conflicting results are likely due to sampling and methodological differences among studies. Also, elevated cortisol concentrations are associated with psychosocial stress (Mayer et al., 2018) and inadequate social support (Iob et al., 2018). Thus, cortisol levels may partly indicate a combined effect of SLE and the participants’ recently received social support.

Saliva and plasma cortisol levels are validated measures of acute stress. Still, they are subject to the normal diurnal variation in cortisol secretion and are easily affected by a psychosocial stressor that occurs right before sample collection (Foley & Kirschbaum, 2010). In contrast, hair cortisol concentration (HCC) reflects long‐term cumulative cortisol secretion and chronic stress response for several months (Manenschijn et al., 2011). On average, human hairs grow 1 cm per month, a hair segment 3 cm long from the scalp can be used to determine the cortisol secretion levels during the preceding 3 months (Wester & van Rossum, 2015). HCC can be a robust biomarker for evaluating stress’ cumulative effects in trauma and stress‐related mental disorders, including schizophrenia and bipolar disorder (Sauve et al., 2007). Studies investigating HCC in schizophrenia and bipolar disorder are scarce, and the results are inconclusive (Aas et al., 2019; Manenschijn et al., 2012; Streit et al., 2016; van den Berg et al., 2020; Yang et al., 2020).

In summary, research findings support the neural diathesis‐stress model that implicates a role for stress in the etiology of schizophrenia and bipolar disorder (Lex et al., 2017; Walker & Diforio, 1997). We have previously shown that HCC is associated with symptoms and social support in schizophrenia, but we have not investigated HCC in bipolar disorder (Yang et al., 2020). Few studies compared the relationship between HCC and psychosocial stress, social support, clinical phenotypes, clinical course, and outcome in schizophrenia and bipolar disorder (Streit et al., 2016). This study aims to investigate and compare the associations between HCC and childhood trauma, the number of SLE, the amount of social support, clinical features, clinical course, and outcome in schizophrenia and bipolar disorder. We seek to clarify to what degree psychosocial stress and social support influence HCC and the association between HCC and clinical features, clinical course, outcome in schizophrenia, and bipolar disorder.

## 2 METHODS

### 2.1 Sample

A total of 109 patients were diagnosed with schizophrenia and 93 patients diagnosed with bipolar disorder were recruited from the Inpatient Department of Shanghai Mental Health Center. Eighty‐six healthy participants were enrolled by means of advertisements from the local community in Shanghai as the control group in the study. The participants were enrolled between January 2018 and December 2019. Inclusion criteria for patients were as follows: (1) between 18 and 60 years old; (2) has a diagnosis of schizophrenia or bipolar disorder according to Diagnostic and Statistical Manual of Mental Disorders (DSM‐IV‐TR) (Association, 2000); (3) a total Positive and Negative Syndrome Scale (PANSS) score between 60 and 120 for schizophrenia patients, and a total Young Mania Rating Scale (YMRS) score ≥ 10 with any Montgomery–Asberg Depression Rating Scale (MADRS) score for bipolar disorder patients at screening.

Inclusion criteria for the healthy controls were (1) between 18 and 60 years old; (2) has no lifetime diagnosis of any psychiatric disorder. Exclusion criteria for all groups included (1) neuroendocrine disorders; (2) neurological disorders; (3) on any medication that may influence HPA axis function; (4) mental disorders due to a known medical illness or substance use; (5) unstable or uncontrolled medical conditions interfering with brain functions; (6) moderate‐to‐severe brain injury; (7) IQ under 70.

Psychiatrists and psychiatrists‐in‐training performed clinical assessments. All clinical personnel completed training in diagnostics and symptoms rating. All participants were interviewed using a pencil‐and‐paper version of the interview on average 1 h for the patients and 30 min for the controls. All participants were informed of the purpose of the study and a written informed consent was established before the interview. The ethics committee in Shanghai Mental Health Center approved this study (registration number 2018‐13).

### 2.2 Measures

PANSS was used to measure the severity of schizophrenia in positive symptoms, negative symptoms, cognitive symptoms, depression–anxiety symptoms, and excitement symptoms (Van den Oord et al., 2006). The severity of manic episode of bipolar disorder was rated using YMRS. The total score ranges from 0 to 60, composed of four items that score between 0 and 8 and seven items that score between 0 and 4 (Young et al., 1978). The severity of depressive episode of bipolar disorder was measured by a 10‐item MADRS that item scores between 0 and 6 (Montgomery & Asberg, 1979). Psychotic symptoms in bipolar disorder were assessed using items adapted from Composite International Diagnostic Interview (CIDI) (Robins et al., 1988). The items include 15 questions for delusions and 6 questions for hallucinations which were rated as absent or present (Cooper et al., 1998). Formal thought disorder was assessed using the Thought, Language, and Communication scale (TLC) (Andreasen, 1986). Briefly, the TLC contains 18 items and an overall rating (global TLC). Severity ratings of the items 1–9 range from 0 (absent) to 4 (extreme), while severity ratings of the items 10–18 range from 0 (absent) to 3 (severe). Personality traits were screened using the Standardized Assessment of Personality–Abbreviated Scale (SAPAS), an 8‐binary question scale that directly refers to an aspect of a person's behavior, emotions, and social relationships. Each answer is scored as either 0 or 1, and the overall score is rated from 0 to 8 (Moran et al., 2003). Neuroticism was measured using the 23‐item Eysenck personality questionnaire (Eysenck, 1975). The Global Assessment of Functioning Scale (GAF) (0 through 100) was used to assess the social, occupational, and psychological functioning during the previous 3 months (Jones et al., 1995).

We used the instrument for SLE assessment employed in the Virginia Adult Twin Study of Psychiatric and Substance Use Disorders (VATSPUD) study, which assessed 13 negative SLE including loss of confidant, marital difficulties, job loss, major financial crisis, legal problems, serious illness, life‐threatening accident, natural disaster, witness of someone being injured or killed, assault, and threat. The time that SLE occurred was recorded if the coding was positive (Kendler & Prescott, 2006). We assessed childhood emotional neglect (CEN), childhood physical abuse (CPA), and childhood sexual abuse (CSA) through the questions adapted from the Childhood Trauma Questionnaire (CTQ) (Bernstein et al., 2003). Sexual abuse refers to any unwanted incidents such as (1) inviting or requesting the child to do something sexual, (2) touching or fondling private parts, (3) making them touch the person in a sexual way, or (4) attempting or having sexual intercourse. Physical abuse refers to bodily assaults on a child by an older person that pose a risk of, or result in, injury. Emotional neglect refers to a lack of emotional support and inadequate attention to a child's emotional needs, including the need for affection.

Social support was measured using the 6‐item short form of the Social Support Questionnaire (SSQ) (Sarason, 1987). SSQ assesses the number of available others the participant can rely on in times of need and how satisfied the participant is with the overall support from the above‐identified people(s) ranging from 1 (Very dissatisfied) to 6 (Very satisfied). The Perceived Stress Scale (PSS) was used to measure the degree to which situations in one's life were appraised as stressful (Cohen et al., 1983). PSS contains 10 items ranging from 0 to 4 (0—never; 1—almost never; 2—sometimes; 3—fairly often; 4—very often). SSQ and PSS were used to evaluate the participant's experiences with stressful events and social support in the last three months.

The age at onset was defined as the age at which the first manifestation of psychotic symptoms or mood symptoms fulfilling the criteria of schizophrenia or bipolar disorder based on DSM‐IV‐TR took place. The illness duration was defined as the time between the age at onset and the research interview time. The risk of relapse was defined as the ratio between number of episodes in lifetime and illness duration. Medication compliance was defined as the proportion of total duration of medicine‐taking throughout the illness. The predominant polarity of bipolar disorder was defined by previous studies as >2:1, that is, at least double the number of episodes of one pole of the disorder over the other (Colom et al., 2015).

### 2.3 Hair sample and hair cortisol analysis

Hair sample preparation was described in detail elsewhere (Yang et al., 2020). A 3‐cm hair segment, approximately 100–150 hairs were cut from the posterior vertex as close to the participant's scalp as possible. The hair samples were stored in envelopes until analysis. As hair grows at an average speed of 1 cm per month, this 3‐cm segment can reflect cortisol secretion in the last 3 months (Gow et al., 2010). The hair sample was minced with small surgical scissors, and 50 mg of powered hair was placed into a glass vial. One milliliter of methanol was added to the vial and was incubated for 16 h at 52℃ with gently shaking in order to extract cortisol. Then, methanol was transferred to a clean glass vial and was evaporated under a constant nitrogen stream until completely dry. The samples were then dissolved in 250 μl of phosphate‐buffered saline (pH 8.0) and vortexed and thoroughly mixed. Cortisol concentrations in the hair extracts were measured using a commercial ELISA kit for salivary cortisol (DRG Instruments GmbH, Marburg, Germany) according to the manufacturer's instructions. Cross reactivity of other steroids with the kit's antibodies was reported as follows: Progesterone (23.40%) and Testosterone (0.04%). Intra‐assay variation was below 6% and the inter‐assay variation was below 7% as reported by the supplier. The analytical sensitivity of the assay is 0.09 ng/ml. HCC value is presented in pg/mg hair.

### 2.4 Statistics

The statistical analyses were completed using R (version 3.3.1) (R Core Team, 2013). The descriptive statistics were presented as percentages for discrete variables and as means (standard deviation, SD) for continuous variables. Chi‐square test and one‐way ANOVA were used to compare the categorical and continuous variables between schizophrenia patients, bipolar disorder patients, and healthy controls. We applied linear regression to examine the associations between HCC, a continuous independent variable, and psychosocial stress, social support, clinical features of schizophrenia and bipolar disorder, including clinical course and outcome. Coefficient values were used to quantify the strength of associations. The statistical significance for all tests was set at *p* < .05 as the analyses were exploratory in nature.

To examine the relationship and factorial constructs of HCC, psychosocial stress, social support, and syndromes in patients, exploratory factor analysis was performed by using both varimax and promax rotations. Interpretations of the scree plot and eigenvalue were used to guide decisions on the number of factors to be extracted. Factor loadings ≧ 0.30 were considered to be substantial.

## 3 RESULTS

The average age of schizophrenia patients, bipolar disorder patients, and healthy controls at interview were 40.8 (SD = 12.2) years, 33.6 (SD = 11.5) years and 42.2 (SD = 10.9) years, respectively (F _2, 285_ = 14.65, *p* < .001). The average age at onset of schizophrenia and bipolar disorder was 27.7 (SD = 11.4) years and 23.46 (SD = 8.32) years (*p* < .05). Bipolar disorder patients have significantly lower HCC (11.3 pg/mg, SD = 7.7) than schizophrenia patients (14.2 pg/mg, SD = 11.7) and healthy controls (18.5 pg/mg, SD = 10.5) (F _2,285_ = 11.25, *p* < .001).

Table 1 shows the results for socio‐demographic features of schizophrenia patients, bipolar disorder patients, and healthy controls. Both schizophrenia patients and bipolar patients were less likely to be employed, to be married and to receive education compared to healthy controls (all *p* < .001).

**TABLE 1:** Socio‐demographic variables between schizophrenia, bipolar disorder, and healthy controls

	Schizophrenia (*N* = 109, %)	Bipolar disorder (*N* = 93, %)	Healthy controls (*N* = 86, %)	Statistics	*p*‐value
Gender					
Male	51 (46.8)	53 (57.0)	38 (44.0)	*X^2 ^* = 3.30	.185
Female	58 (53.2)	40 (43.0)	48 (56.0)		
Marital status					
Married	45 (41.3)	39 (41.9)	74 (86.0)	*X^2 ^* = 50.62	**<.001**
Separated/divorced	12 (11.0)	11 (11.8)	0		
Widowed	1 (0.9)	0	0		
Never married	51 (46.8)	43 (46.2)	12 (14.0)		
Education					
Primary school	5 (4.6)	1 (1.1)	6 (7.0)	*X^2 ^* = 45.15	**<.001**
High school	39 (35.8)	27 (29.0)	30 (35.0)		
Technical school	40 (36.7)	31 (33.3)	6 (7.0)		
Bachelor	24 (22.0)	32 (34.4)	32 (37.0)		
Master or PhD	1 (0.9)	2 (2.2)	12 (14.0)		
Employment					
Working for pay	16 (14.7)	48 (51.6)	74 (86.0)	*X^2 ^* = 123.88	**<.001**
Retired	28 (25.7)	10 (10.8)	2 (2.0)		
Laid off and looking for work	56 (51.4)	20 (21.5)	0 (0)		
Keeping house	2 (1.8)	5 (5.4)	4 (5.0)		
Other	7 (6.4)	10 (10.8)	6 (7.0)		

The differences of SLE, childhood trauma, social support, perceived stress, neuroticism, personality traits, and body mass index between schizophrenia patients, bipolar patients, and healthy controls are presented in Table 2. Compared with healthy controls, schizophrenia patients and bipolar patients had more SLE in lifetime (*p* < .01), fewer people whom they could count on to be consoled (*p* < .01) and a smaller number of available others (*p* < .05). Schizophrenia patients had fewer people who totally accepted them than healthy controls had (*p* < .05).

**TABLE 2:** Comparisons of variables between different groups

Variables	Schizophrenia patients (*N* = 109, mean, SD)	Bipolar disorder patients (*N* = 93, mean, SD)	Healthy controls (*N* = 86, mean, SD)	Statistics	*p*‐value
SLE in lifetime	1.37 (2.83)*	1.84 (2.40)**	0.60 (0.88)	*F*_2,285_ = 6.81	**.001**
SLE in recent 3 months	0.04 (0.19)	0.08 (0.27)	0.02 (0.15)	*F*_2,285_ = 1.93	.150
CEN	0.16 (0.77)	0.08 (0.40)	0.02 (0.15)	*F*_2,285_ = 1.70	.184
CPA	0.09 (0.59)	0.16 (0.73)	0.14 (0.35)	*F*_2,285_ = 0.39	.678
CSA	0.06 (0.50)	0.17 (0.76)	0	*F*_2,285_ = 2.39	.093
Number of available others	1.87 (1.23)*	2.01 (1.15)*	2.38 (1.21)	*F*_2,285_ = 4.51	**.012**
Number of people count on to be dependable	2.43 (1.48)	2.93 (2.0)	2.91 (1.84)	*F*_2,285_ = 2.61	.076
Number of people count on to help you feel more relaxed	1.76 (1.54)	2.14 (1.94)	2.10 (1.32)	*F*_2,285_ = 1.69	.187
Number of people accepts you totally	1.75 (1.22)*	2.07 (1.54)	2.23 (1.19)	*F*_2,285_ = 3.37	.036
Number of people count on to care about you	2.01 (1.24)	2.17 (1.30)	2.14 (1.13)	*F*_2,285_ = 0.49	.614
Number of people count on to help you feel better	1.84 (1.35)	2.08 (1.66)	2.23 (1.38)	*F*_2,285_ = 1.77	.172
Number of people count on to console you	1.71 (1.28)**	1.9 (1.48)*	2.53 (2.27)	*F*_2,285_ = 5.97	**.003**
PSS	14.50 (7.90)	12.6 (9.30)	12.0 (6.2)	*F*_2,285_ = 2.70	.069
EPQ	6.60 (5.60)**	6.70 (5.70)**	2.95 (3.29)	*F*_2,285_ = 16.10	**<.001**
Personality traits score	2.65(1.63)**	3.12 (1.51)**,^∆^	0.95 (1.02)	*F*_2,285_ = 56.65	**<.001**
Detachment personality factor	1.18 (0.80)**	0.57 (0.77)** ^,^ ^∆∆^	0.26 (0.49)	*F*_2,285_ = 42.84	**<.001**
Externalizing personality factor	0.39 (0.63)*	1.05 (0.85)** ^,^ ^∆∆^	0.19 (0.39)	*F*_2,285_ = 43.33	**<.001**
Negative affectivity personality factor	1.08 (0.93)**	1.50 (1.01)** ^,^ ^∆∆^	0.51 (0.67)	*F*_2,285_ = 27.82	**<.001**
BMI	23.29 (3.67)	23.54 (3.38)	23.18 (2.55)	*F*_2,285_ = 0.29	.750

*Abbreviations*: BMI, body mass index; CEN, childhood emotional neglect; CPA, childhood physical abuse; CSA, childhood sexual abuse; EPQ, Eysenck Personality Questionnaire; PSS, Perceived Stress Scale; SD, standard deviation; SLE, stressful life events.

* *p* < .05.

** *p* < .01, compared with healthy controls.

^∆^ *p* < .05.

^∆∆^ *p *< .01, compared with schizophrenia patients.

Schizophrenia patients and bipolar patients had a significantly higher neuroticism score than healthy controls (F _2,285_ = 16.10, *p* < .001). Bipolar patients had the highest personality traits score. The healthy controls had the lowest personality traits score, while the personality traits score of the schizophrenia patients was in the middle (F _2,285_ = 56.65, *p* < .001). Furthermore, schizophrenia patients showed the highest score on detachment personality traits (e.g., a loner), and bipolar patients showed highest score on both externalizing personality traits (e.g., losing temper easily) and negative affectivity personality traits (e.g., a worrier). There are significantly higher rates of personality traits both in the bipolar group (62.1%) and schizophrenia group (51.5%) compared to the healthy control group (6.9%) (*p* < .001, Table 2).

We examined the association between HCC and variables including psychosocial stress, social support, perceived stress, neuroticism, personality traits, and body mass index in schizophrenia patients, bipolar disorder patients, and healthy controls (Table 3). In the schizophrenia patients, HCC was positively associated with the number of available others (*p* = .02), including the number of people they can count on to be dependable (*p* = .01) and count on to be cared about (*p* = .01). HCC was also positively associated with personality traits (*p* = .003) and detachment personality traits (*p* = .001). In the healthy controls, HCC was only associated with detachment personality traits (*p* = .04). There were no significant associations between HCC and the above variables in bipolar disorder patients.

**TABLE 3:** Association between HCC and variables in different groups

	Schizophrenia (*N* = 109)		Bipolar disorder (*N* = 93)		Healthy controls (*N* = 86)	
Variables	Coefficient ± *SE*	*p*	Coefficient ± *SE*	*p*	Coefficient ± *SE*	*p*
SLE in lifetime	−1.88 ± 1.08	.08	−0.57 ± 0.50	.26	0.88 ± 1.87	.64
SLE in recent 3 months	−3.01 ± 5.99	.62	−3.46 ± 3.29	.30	0.00 ± 10.72	1.00
CEN	−0.37 ± 2.07	.86	−1.22 ± 3.52	.73	−5.73 ± 10.68	.60
CPA	−3.46 ± 5.90	.56	−0.47 ± 1.36	.73	−1.45 ± 4.67	.76
CSA	−3.73 ± 8.39	.66	−1.26 ± 1.25	.32	NA	NA
Number of available others	**3.38 ± 1.40**	**.02**	−0.49 ± 0.97	.61	−0.86 ± 1.36	.53
Number of people count on to be dependable	**2.28 ± 0.87**	**.01**	−0.66 ± 0.48	.17	−0.74 ± 0.89	.41
Number of people count on to help you feel more relaxed	1.75 ± 0.96	.07	−0.04 ± 0.52	.94	−0.80 ± 1.25	.52
Number of people accepts you totally	1.01 ± 1.14	.38	−0.04 ± 0.62	.95	−1.97 ± 1.34	.15
Number of people count on to care about you	**2.62 ± 1.03**	**.01**	−0.52 ± 0.71	.47	0.41 ± 1.45	.78
Number of people count on to help you feel better	1.10 ± 1.03	.29	−0.18 ± 0.68	.79	−0.41 ± 1.19	.73
Number of people count on to console you	1.65 ± 1.12	.15	−1.20 ± 0.64	.07	0.15 ± 0.72	.83
PSS	0.21 ± 0.22	.33	0.03 ± 0.12	.80	0.01 ± 0.27	.99
EPQ	0.53 ± 0.29	.07	0.02 ± 0.16	.91	−0.29 ± 0.50	.56
Personality traits score	2.97 ± 0.97	**.003**	0.45 ± 0.80	.58	−2.38 ± 1.58	.14
Detachment personality factor	6.66 ± 1.97	**.001**	1.56 ± 1.47	.30	0.26 ± 0.49	**.04**
Externalizing personality factor	3.11 ± 2.87	.28	1.02 ± 1.47	.49	−3.58 ± 4.12	.39
Negative affectivity personality factor	2.72 ± 1.71	.12	−0.68 ± 1.21	.57	−0.77 ± 2.46	.76
BMI	−0.10 ± 0.34	.77	−0.10 ± 0.29	.77	0.47 ± 0.64	.47

*Abbreviations*: BMI, body mass index; CEN, childhood emotional neglect; CPA, childhood physical abuse; CSA, childhood sexual abuse; EPQ, Eysenck Personality Questionnaire; HCC, hair cortisol concentration; PSS, Perceived Stress Scale; SE, standard error; SLE, stressful life events.

*p < .05.

The differences in clinical features, including symptoms, thought disorder, course characteristic (age at onset, illness duration, risk of relapse), medication compliance, and social functioning between schizophrenia patients and bipolar disorder patients, are presented in Table 4. The depression–anxiety factor score and excitement factor score accounted for 13.7% and 13.2% of the PANSS total score in the schizophrenia patients. While 73.2% of the bipolar disorder patients reported psychotic symptoms, 65.2% of them had a predominant polarity and 81.7% of them were with bipolar disorder I. Bipolar disorder patients had an earlier age at onset (*p* = .004), higher risk of relapse (*p* = .007) and more episodes during their lifetime (*p* < .001) compared to schizophrenia patients. Yet, bipolar disorder patients’ social functioning was better than that of schizophrenia patients’ (*p* < .001). Bipolar disorder patients did not differ from schizophrenia patients in terms of the illness duration, medication compliance, and the severity of thought disorder (all *p* > .05).

**TABLE 4:** Descriptions and comparisons of clinical features between schizophrenia and bipolar disorder

Clinical features	Schizophrenia patients (*N* = 109, Mean, SD or %)		Bipolar disorder patients (*N* = 93, Mean, SD or %)		Statistics	*p*
Symptom severity	PANSS total score	75.29 (10.12)	YMRS score	35.89 (10.60)		
	Positive symptoms factor	18.27 (3.93)	MADRS score	32.7 (10.93)		
	Negative symptoms factor	16.40 (5.36)	Psychotic bipolar disorder	73.2%		
	Cognitive factor	14.14 (5.21)	Non‐psychotic bipolar disorder	16.8%		
	Depressive anxiety factor	10.30 (3.71)				
	Excitement factor	9.84 (4.13)				
Course characteristic	Multiple episodes, currently in acute episode	82.6%	NPP	34.8%		
	First episode, currently in acute episode	14.7%	DPP	22.7%		
	Continuous	2.7%	MPP	42.5%		
TLC total score		10.06 (9.11)		9.36 (7.19)	*t* = −0.60	.55
TLC overall rating		1.43 (0.98)		1.27 (0.94)	*t *= −1.18	.24
Illness duration, years		13.15 (12.51)		10.61 (8.62)	*t* = −1.65	.10
Total episodes (*N*)		3.76 (2.75)		5.41 (3.07)	*t* = 4.03	**<.001**
Risk of relapse		0.69 (0.65)		0.97 (0.82)	*t* = 2.71	**.007**
Medication compliance (%)		59.37 (33.49)		55.26 (30.67)	*t* = −0.90	.37
GAF score		76.66 (11.22)		86.7 (4.44)	*t* = 8.10	**<.001**

*Abbreviations*: DPP, depressive predominant polarity; GAF, global assessment of functioning scale; MADRS, Montgomery–Asberg Depression Rating Scale; MPP, manic predominant polarity; NPP, non‐predominant polarity; PANSS, Positive and Negative Syndrome Scale; SD, standard deviation; TLC, Thought, Language, and Communication Scale; YMRS, Young Mania Rating Scale.

We applied linear regression to explore the association between HCC and a series of clinical features in schizophrenia and bipolar disorder. The results are presented in Table 5. HCC was positively associated with the severity of depression–anxiety factor in PANSS score in schizophrenia patients (*p* = .03) and negatively associated with the severity of thought disorder in bipolar disorder patients (*p* = .04). No other significant associations were found between HCC and illness duration, total episodes, risk of relapse, medication compliance, and social functioning of schizophrenia and bipolar disorder patients.

**TABLE 5:** Association between HCC and clinical features in schizophrenia and bipolar disorder

Clinical features	Schizophrenia patients (*N* = 109, coefficient ± *SE*)		*p*	Bipolar disorder patients (*N* = 93, coefficient ± *SE*)		*p*
Symptom severity	PANSS total score	0.05 ± 0.12	.68	YMRS score	–0.07 ± 0.09	.43
	Positive symptoms factor	–0.36 ± 0.30	.23	MADRS score	0.04 ± 0.10	.65
	Negative symptoms factor	–0.10 ± 0.22	.65	Psychotic bipolar disorder	0.18 ± 0.98	.85
	Cognitive factor	–0.03 ± 0.23	.89	Non‐psychotic bipolar disorder		
	Depressive anxiety factor	0.68 ± 0.32	**.03**			
	Excitement factor	0.35 ± 0.30	.25			
Illness course character	Multiple episodes/First episode/Continuous	0.31 ± 0.75	.75	NPP/DPP/MPP	0.91 ± 1.49	.54
TLC total score		0.09 ± 0.15	.57	–0.26 ± 0.13		**.04**
TLC overall rating		0.99 ± 1.29	.45	–1.39 ± 1.02		.17
Illness duration (years)		–0.04 ± 0.10	.71	–0.13 ± 0.16		.43
Total episodes (*N*)		–0.07 ± 0.44	.88	–0.24 ± 0.40		.56
Risk of relapse		2.09 ± 2.02	.30	0.54 ± 1.46		.71
Medication compliance (%)		0.02 ± 0.05	.71	–0.06 ± 0.06		.27
GAF score		0.15 ± 0.13	.25	0.32 ± 0.35		.37

*Abbreviations*: DPP, depressive predominant polarity; GAF, global assessment of functioning scale; HCC, hair cortisol concentration; MADRS, Montgomery–Asberg Depression Rating Scale; MPP, manic predominant polarity; NPP, non‐predominant polarity; PANSS, Positive and Negative Syndrome Scale; SE, standard error; TLC, Thought, Language, and Communication Scale; YMRS, Young Mania Rating Scale.

Exploratory factor analysis was employed to examine and compare the relationship between psychological stress, social support, clinical features, social functioning, and HCC in schizophrenia and bipolar disorder. Examination of the scree plot and eigenvalue indicated that a four‐factor solution best fitted the data. We examined both an orthogonal and oblique factor rotation which produced similar results. The orthogonal rotations are more likely to approximate clinical reality. Factor analysis of the 11 or 12 items yielded four factors accounting for 59% of the variance in schizophrenia and 54% of the variance in bipolar disorder (Table 6A,B). Factor analysis results show that schizophrenia and bipolar disorder share a very similar (factor I to III) but somewhat different (factor IV) factor structure for psychosocial stress, social support, clinical features, social functioning, and HCC.

**TABLE 6:** Comparison of exploratory factor analysis of variables between schizophrenia and bipolar disorder

Table 6A				
	Factor I	Factor II	Factor III	Factor IV
Stressful life events	0.99			
Childhood physical abuse	0.84			
Childhood emotional neglect	0.74			
Childhood sexual abuse	0.90			
Neuroticism		0.98		
Perceived stress scale		0.67		
Cognitive factor in PANSS			0.47	
Thought disorder			0.99	
Social support				0.60
Hair cortisol concentrations				0.44
Global assessment function				

Table 6B				
	Factor I	Factor II	Factor III	Factor IV
Stressful life events	0.88			
Childhood physical abuse	0.88			
Childhood emotional neglect	0.88			
Childhood sexual abuse	0.76	0.31		
Neuroticism		0.87		–0.44
Perceived stress scale	0.36	0.60		
Young Mania Rating Scale score			0.59	
Thought disorder			0.97	
Social support				
Hair cortisol concentrations				
Global assessment function				0.37
Montgomery–Asberg Depression Rating Scale score				

*Note*: Data indicates factor loadings for orthogonal rotated solution.

Items within a factor are supposed to be interrelated. In both schizophrenia and bipolar disorder, SLE, CPA, CEN, and CSA loaded prominently on factor I. Neuroticism and perceived stress loaded substantially on factor II. Symptoms (cognitive symptoms in schizophrenia and manic symptoms in bipolar disorder) and thought disorder loaded the highest on factor III. HCC and social support are positively correlated and loaded substantially on factor IV in schizophrenia. Social functioning and neuroticism are inversely correlated and loaded prominently on factor IV in bipolar disorder.

## 4 DISCUSSION

Our study aims to examine and compare the relationship between HCC and childhood trauma, the number of SLE, the amount of social support, clinical features, clinical course, and outcome in schizophrenia and bipolar disorder. Our results show the risk of relapse is higher in bipolar disorder patients than in schizophrenia patients despite the similar number of experienced psychosocial stressors, the amount of received social support, and medication compliance. We find HCC is decreased significantly in both schizophrenia patients and bipolar disorder patients compared to healthy controls. HCC is significantly associated with clinical syndromes, including depression–anxiety factor of PANSS in schizophrenia patients and thought disorder in bipolar disorder patients. However, HCC is positively associated with social support and personality traits only in schizophrenia, but not in bipolar disorder patients. Factor analysis indicates schizophrenia and bipolar disorder share a very similar but somewhat different structure in terms of HCC, psychosocial stress, social support, clinical features, clinical course, and outcome.

Consistent with previous studies, patients with schizophrenia and bipolar disorder are more likely to have experienced intense psychosocial adversities (Mauritz et al., 2013; Watson et al., 2014) and have received less social support than healthy controls (Munikanan et al., 2017; Xie et al., 2018). Increased subjective social support is correlated with a lower degree of psychotic symptoms (Peng et al., 2019) and higher recovery scores (Dunne et al., 2019). On the contrary, exposure to psychosocial stress is strongly associated with onset of psychosis in schizophrenia and bipolar disorder (Green et al., 2014; Holtzman et al., 2013). Few studies compare the psychosocial stress and social support between schizophrenia and bipolar disorder directly (Laursen & Munk‐Olsen, 2007). We found no significant differences between schizophrenia and bipolar disorder in terms of the number of experienced psychosocial stressors and the amount of received social support. A large‐scale study has shown there is an overlap in the environmental risk factors for schizophrenia and bipolar disorder. The differences between the two major psychiatric disorders are quantitative rather than qualitative, which suggests a genetic and environmental overlap between the disorders (Laursen & Munk‐Olsen, 2007).

Yet, both the risk of relapse (more episodes per year) and GAF score (better social functioning) are higher in the bipolar disorder patients than in schizophrenia patients despite the similar number of experienced psychosocial stressors, the amount of received social support, illness duration, and medication compliance. This finding agrees with other studies reporting significantly higher rates of relapses and hospitalizations among patients with bipolar disorder than among those with schizophrenia (Ayano & Duko, 2017; Finseth et al., 2014). Our results support the widely accepted concept of the recurrent clinical course of bipolar disorder and the chronic fluctuating clinical course of schizophrenia (Aykut et al., 2017). Factors influencing the risk of relapse of schizophrenia and bipolar disorder include SLE, social support, the severity of episodes, alcohol, female gender, residual symptoms at recovery, medication, and compliance (Morken et al., 2008; Perlis et al., 2006). The combined effects of risk factors (e.g., childhood adversities, expressed emotions, etc.) and protective factors (e.g., antipsychotic medications, family interventions) might contribute to the symptomatic relapses in schizophrenia and bipolar disorder (Cohen et al., 2004; Lecomte et al., 2019). Besides the risk and protective factors mentioned above, more previous episodes predict increased minor events and episodes in bipolar disorder, which indicate a sensitization effect of psychosocial stress mainly on the clinical course of bipolar disorder (Weiss et al., 2015).

In line with other studies, our findings show more personality traits in schizophrenia and bipolar disorder patients compared to healthy controls, including higher level of neuroticism, externalizing personality traits, emotional instability, and detachment personality traits (Lysaker & Taylor, 2007; Solomon et al., 1996). We find different patterns of personality traits between schizophrenia patients and bipolar disorder patients. The former scores highest on detachment personality traits, while the latter scores highest on externalizing and negative affectivity personality traits. Other studies report personality traits differ (Lonnqvist et al., 2009) or do not differ between schizophrenia and bipolar disorder patients (Wilson & Sponheim, 2014). Personality traits appear to be dimensional and could predict future onset of schizophrenia (neuroticism), bipolar disorder (high extraversion) or psychosis (schizotypal) (Lonnqvist et al., 2009; Urosevic et al., 2019). The interaction between the environmental risk factors (e.g., stress), personality, and genetic predisposition may contribute to the multifactorial causation of schizophrenia and bipolar disorder (Agid et al., 1999).

Consistent with other studies, our results show schizophrenia and bipolar disorder are characterized by substantial clinical overlap, including the psychiatric syndromes (Clementz et al., 2020). In our sample, 73.2% of the bipolar disorder patients reported psychotic symptoms. The average depression–anxiety factor score and excitement factor score accounts for 26.7% of the total PANSS score. The severity of thought disorder between bipolar disorder patients and schizophrenia patients is comparable. The clinical phenotype of schizophrenia mainly includes delusions, hallucinations, bizarre or disorganized behavior and negative symptoms. However, depressive and hypomanic symptoms are also observed during the clinical course (Jager et al., 2008). Bipolar disorder refers to periodic changes in mood and activity from depression, hypomania to mania, whereas psychotic symptoms (hallucinations and delusions) also appear in bipolar disorder (Allardyce et al., 2007; Henry & Etain, 2010). Five factors have been identified (mania, reality distortion, depression, disorganization, negative) both in schizophrenia and bipolar disorder. All are more variable in schizophrenia than in bipolar disorder. Mania is the best discriminator between schizophrenia and bipolar disorder, while the negative factor is strongly correlated with poor premorbid functioning, insidious onset, and worse course (Dikeos et al., 2006).

Studies report mixed results on the cortisol concentrations in schizophrenia and bipolar disorder, including elevated (Ellenbogen et al., 2011; Steen et al., 2014), normal (Deshauer et al., 2006; Girshkin et al., 2016) or decreased (Hempel et al., 2010) serum or salivary cortisol concentrations compared to healthy controls. Significant differences or no differences of serum and salivary cortisol levels between schizophrenia and bipolar disorder have been reported (Girshkin et al., 2014). HCC reflects long‐term cumulative cortisol secretion over weeks to months and studies investigating HCC in schizophrenia and bipolar disorder are scarce (Aas et al., 2019; Streit et al., 2016; Yang et al., 2020). Our results show schizophrenia patients and bipolar disorder patients experience more SLE and less social support in their lifetime, and they have lower HCC compared to healthy controls. HCC in bipolar disorder patients is significantly lower than those in schizophrenia patients. However, other studies report higher or normal HCC in schizophrenia and bipolar disorder patients relative to healthy controls, (Aas et al., 2019; Manenschijn et al., 2012; Streit et al., 2016) and bipolar disorder patients have higher or similar HCC compared to schizophrenia patients (Aas et al., 2019; Streit et al., 2016).

Psychosocial stress (childhood maltreatment or SLE) and social support seem to be associated with cortisol concentrations both in schizophrenia and bipolar disorder. The latter attenuate the effects of SLE on cortisol concentrations (Iob et al., 2018; Tas et al., 2018). We find HCC is positively associated with social support in schizophrenia patients but not in bipolar disorder patients. Other confounding factors influencing cortisol levels in schizophrenia and bipolar disorder include psychotropic medication, alcohol use, gender, BMI and blood pressure, and so forth (Stalder et al., 2017; Subramaniam et al., 2019; Zhang et al., 2005). The factors mentioned above might contribute to the inconsistent findings across different studies. Despite these conflicting findings, results support the neural stress‐diathesis model, which elaborates the role of stress and HPA axis dysfunction in the etiology of schizophrenia and bipolar disorder (Pruessner et al., 2017; Streit et al., 2016).

Cortisol concentrations are positively or negatively associated with the severity of negative symptoms, positive symptoms, hopeless, neurocognitive function, or the severity of a wide array of schizophrenia and bipolar disorder symptoms (Tournikioti et al., 2018; Zhang et al., 2005). Our results align with the previous findings and show HCC is associated with clinical syndromes in schizophrenia (depression–anxiety factor in PANSS) and bipolar disorder (thought disorder). Glucocorticoid receptors are present throughout the central nervous system and thus can mediate the effects of cortisol on several neural systems in several brain areas (Murri et al., 2016; McEwen, 2007). The synergistic relation between HPA activity and monoamines neurotransmission (DA, NE, 5‐HT) can help us to understand how stress exposure leading to increased cortisol secretion might trigger a neuropathological process (Schifani, Hafizi, et al., 2019).

Factor analysis in our study reveals four latent factors within schizophrenia and bipolar disorder. The first factor consists of psychosocial stress, including both childhood trauma and adulthood SLE. The second factor includes thought disorder and different syndromes in schizophrenia (cognitive dysfunction) and bipolar disorder (mania). The third factor includes perceived stress and neuroticism. The fourth factor stands for the differences between schizophrenia and bipolar disorder in terms of HCC, social support, and social functioning. Hence our data suggest a very similar factor structure in schizophrenia and bipolar disorder in terms of the psychosocial stress, social support, perceived stress, and clinical phenotype. Schizophrenia and bipolar disorder have a significant overlap both in environmental risk factors and clinical phenotype.

Our results corroborate the previous findings about the relationship between schizophrenia and bipolar disorder and shed light on the HCC as one of the biomarkers for schizophrenia and bipolar disorder (Dikeos et al., 2006; Schifani , Pruessner, et al., 2019). Although HCC predicts the severity of clinical syndromes at certain extent, it does not predict the course and outcome for schizophrenia and bipolar disorder. This might indicate the clinical course and outcome of schizophrenia and bipolar disorder are independent of HCC, perhaps they are also independent of psychosocial stress (Dienes et al., 2006; Lewandowski et al., 2011). HCC is associated with clinical syndromes in both bipolar disorder and schizophrenia, but only associated with social support, personality traits in schizophrenia. Compared to bipolar disorder, schizophrenia seems to be the more severe psychiatric disorder in which more clinical variance and more biological determinants have been involved, including HPA dysfunction (Steen et al., 2014).

This study has several strengths. All of the clinical data were collected through face‐to‐face interviews by trained interviewers with clinical backgrounds. To our knowledge, it is the first study to examine and compare the relationship between psychosocial stress, social support, clinical features, and HCC in Han Chinese schizophrenia and bipolar disorder patients.

This study has a number of limitations which should be carefully considered. First, this is a cross sectional study: data were collected retrospectively and recall bias will have affected results. Second, no causal conclusions can be drawn because all the variables were measured only once. Third, we only assessed the significant negative SLE, such as the death of significant others and job loss, in our study. The influence of everyday living from daily hassles and uplifts which can be measured using the Hassles and Uplifts Scale, was not assessed (DeLongis et al., 1988). We could not rule out the confounding effects of the above unassessed psychological factors on HPA axis (Ravindran et al., 1996). Fourth, we did not examine detailed information on medication for this sample. Thus, we were not able to adjust for potential effect of medication on HPA axis. Antipsychotic medication may increase (Jakovljevic et al., 2007), decrease (Tanaka et al., 2008), or have no influence on cortisol levels in schizophrenia patients (Erjavec et al., 2017). And lastly, previous research suggests that cortisol secretion is attenuated during summer and elevated during winter (Persson et al., 2008). Since all the participants are enrolled from January 2018 and December 2019, the seasonal variation of hair cortisol could have already influenced the results. Thus, this may contribute to the inconsistencies of HCC between our study and the others.

Our results corroborate that schizophrenia and bipolar disorder are psychiatric disorders with distinct as well as overlapping environmental risk factors and clinical characteristics. Although HCC is associated with the severity of clinical syndrome in both schizophrenia and bipolar disorder, it does not predict the course of schizophrenia and bipolar disorder. However, HCC is only associated with social support and comorbid personality traits in schizophrenia, but not in bipolar disorder. Aberrant HCC is related to the syndrome severity of both schizophrenia and bipolar disorder, particularly contributing to the complexity of clinical characteristics in schizophrenia.

## CONFLICT OF INTEREST

On behalf of all the authors, the corresponding authors state that there is no conflict of interest.

## AUTHOR CONTRIBUTIONS

Conceived and designed the experiments: Fuzhong Yang and Hua Xiao. Performed the experiments: Fuzhong Yang, Xiangfei Hong, Jing Tao, and Yupeng Chen. Analyzed the data: Fuzhong Yang. Wrote the paper: Fuzhong Yang. Revised the draft of the manuscript: Yanbo Zhang. All authors have read and approved the final article.

## Data Availability

The data that support the findings of this study are available from the corresponding authors upon reasonable request.
